# Association Between Atopic Keratoconjunctivitis and the Risk of Recurrent Corneal Erosion

**DOI:** 10.3389/fmed.2021.688355

**Published:** 2021-06-02

**Authors:** Ren-Long Jan, Shih-Feng Weng, Jhi-Joung Wang, Sung-Huei Tseng, Yuh-Shin Chang

**Affiliations:** ^1^Department of Pediatrics, Chi Mei Medical Center, Liouying, Tainan, Taiwan; ^2^Graduate Institute of Medical Science, College of Health Science, Chang Jung Christian University, Tainan, Taiwan; ^3^Department of Healthcare Administration and Medical Informatics, Kaohsiung Medical University, Kaohsiung, Taiwan; ^4^Department of Medical Research, Chi Mei Medical Center, Tainan, Taiwan; ^5^Department of Anesthesiology, Chi Mei Medical Center, Tainan, Taiwan; ^6^AI Biomed Center, Southern Taiwan University of Science and Technology, Tainan, Taiwan; ^7^Department of Ophthalmology, National Cheng Kung University Hospital, College of Medicine, National Cheng Kung University, Tainan, Taiwan; ^8^Department of Ophthalmology, Chi Mei Medical Center, Tainan, Taiwan

**Keywords:** atopic keratoconjunctivitis, recurrent corneal erosion, Taiwan Longitudinal Health Insurance Database, cohort study, hazard ratio

## Abstract

**Purpose:** To investigate the risk of recurrent corneal erosion (RCE) in patients with atopic keratoconjunctivitis (AKC).

**Methods:** This national, retrospective, matched cohort study enrolled 184,166 newly-diagnosed AKC patients, selected from the Taiwan National Health Insurance Research Database and identified by the International Classification of Diseases, Ninth Revision, Clinical Modification (ICD-9-CM) code 372.05. The control group comprised 184,166 non-AKC patients matched by age, sex, and potential comorbidities and they were selected from the Taiwan Longitudinal Health Insurance Database, 2000. Information from patients was gathered from 1 January 2004 to 31 December 2011, and both groups were traced from the index date until December 2013. The incidence and risk of RCE (ICD-9-CM code 361.42) was compared between the groups. The adjusted hazard ratio (HR) for RCE was obtained by a Cox proportional hazard regression analysis. The Kaplan–Meier analysis was performed to calculate the cumulative incidence of RCE.

**Results:** In total, 564 AKC patients and 406 non-AKC controls developed RCE during the follow-up span. The incidence of RCE was 1.45 times higher in AKC patients than in controls (95% confidence interval [CI] = 1.27–1.64; *P* < 0.0001). After adjusting for potential confounders, including diabetes mellitus, keratoconjunctivitis sicca, corneal transplantation, ocular blunt trauma, corneal dystrophy, and band keratopathy, AKC patients were 1.36 times more likely to develop RCE than controls (adjusted HR, 1.36; 95% CI = 1.19–1.54; *p* < 0.05).

**Conclusions:** AKC Patients had an increased risk of developing RCE and should be informed of this risk.

## Introduction

Atopic keratoconjunctivitis (AKC), a chronic, non-infectious inflammatory ocular surface situation, is the most severe condition of allergic conjunctival disease. The disease is well-known as an ocular complication with atopic diseases. The symptoms of patients with AKC include itching, redness, tearing, pain in the eyes, and blurred vision (1, 2). Patients with AKC often present characteristics such as eyelid thickening and oedema, conjunctival congestion and thickening, tear film instability and dysfunction, and corneal scarring and neovascularization (2, 3). The pathophysiology of AKC includes eosinophil-associated inflammation, cytokine-mediated immune reactions, and immunoglobulin E-mediated mast cell degranulation (2, 4).

Recurrent corneal erosion (RCE), a relative common disorder worldwide, is characterized by recurrent detachment of the corneal epithelium from the basement membrane. The most frequent clinical presentation of RCE is sudden onset of eye pain accompanied by associated symptoms including photophobia, redness and tearing (5, 6). Corneal epithelial basement membrane dystrophies and mechanical or surgical trauma to the corneal epithelium are important risk factors for RCE (5, 7). Inflammation related to corneal surface injury results in weakening of the extra-cellular adhesion network and disruption of the basement membrane (8, 9).

Eosinophils and their toxic products may play important roles in ocular surface complications including persistent corneal epithelial defects or ulceration in patients with AKC (10, 11). Chronic eye rubbing, a common finding in AKC patients, may lead to ocular surface injury and inflammatory processes, which are well-known risk factors for RCE. Furthermore, up-regulation of matrix metalloproteinase (MMP) is found in both, patients with AKC and patients with RCE (12–14). Consequently, it is clinically relevant to survey whether AKC poses a risk for RCE.

To the best of our knowledge, no previous large-scale cohort studies have examined whether AKC poses a risk for subsequent RCE. A few studies have discussed whether AKC is a contribution factor for RCE, but these were limited to small-scale case series and case reports (10, 11). Hence, we designed a nationwide, population-based cohort study to investigate the risk of RCE in patients with AKC in Taiwan.

## Materials and Methods

### Database

Since March 1, 1995, a single-payer National Health Insurance (NHI) scheme has been running, which has provided all residents with extensive medical care coverage in Taiwan. According to the data compiled after 2007, more than 98% of the total Taiwanese population, ~22.60 million individuals, were enrolled in this program.

Data used in this study were provided from the National Health Insurance Research Database (NHIRD). The Taiwan National Health Research Institute (NHRI) constructed NHIRD, which records coded information on each enrolee's demographics such as birthday, sex, residential area, as well as the International Classification of Diseases, Ninth Revision, Clinical Modification (ICD-9-CM) codes of diagnoses, prescriptions, procedures, and expenditures, irrespective of whether the patient underwent hospitalization or was under ambulatory care. The database is released by the Taiwan NHRI to the public through a formal application. The Institutional Review Board of the Chi Mei Medical Center waived the ethical approval and informed consent as no personally identifiable information was investigated in this database.

### Selection of Patients and Variables

**Figure 1:**
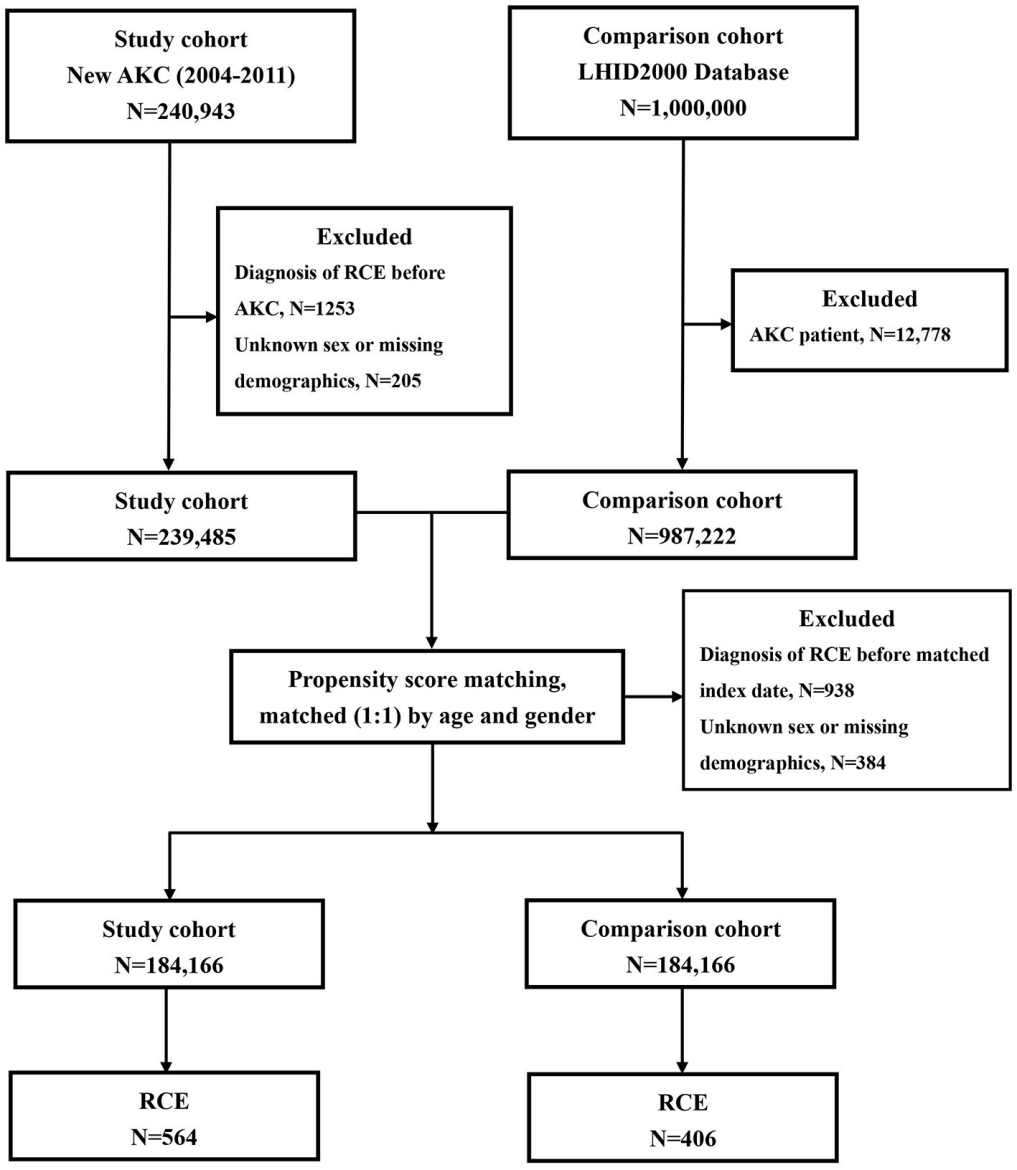
The flowchart for patients with atopic keratoconjunctivitis (AKC) and controls enrollment.

A total of 184,166 newly-diagnosed AKC patients with the ICD-9-CM code 372.05 were recruited for this retrospective cohort study. We collected patient information for the period from 1 January 2004 to 31 December 2011. Figure 1 shows the flowchart of our study. Initially, we included 240,493 patients with a diagnosis of AKC (ICD-9-CM code 372.05). In total, 1,253 subjects diagnosed with RCE (ICD-9-CM code 361.42) before AKC diagnosis and 205 patients with unknown sex or other missing demographic data were excluded.

For each AKC patient, one non-AKC control was randomly selected from the Longitudinal Health Insurance Database 2000 (LHID 2000), a portion of the NHIRD. LHID 2000 includes the entire claims data for 1,000,000 beneficiaries from the year 2000. Initially, 12,778 subjects who had already been diagnosed with AKC, among the 1,000,000 subjects of the LHID 2000, were excluded; thereafter, additional 938 subjects diagnosed with RCE (ICD-9-CM code 361.42) before the index date and 384 patients with unknown sex or missing demographic data were excluded. The 184,166 controls without AKC were matched with the patients with AKC for age (±30 days), sex, and the index date. The index date for AKC patients was defined as the date of diagnosis of AKC; the index date for the control patients was matched with that of the AKC subjects.

To determine the incidence of RCE, we traced the participants in both groups and recorded demographic data of each participant from the index date until death or the end of 2013, whichever occurred earlier. Furthermore, data regarding risk factors, such as comorbidities, including diabetes mellitus (ICD-9-CM code 250) and keratoconjunctivitis sicca (ICD-9-CM codes 370.33 and 710.2); band keratopathy (ICD-9-CM code 371.43); corneal dystrophy (ICD-9-CM code 371.5); corneal transplantation (order codes 85212B, 85213B, 85215B, 85216B, and 85217B); and ocular blunt trauma (ICD-9-CM codes 921.1, 921.2, 921.3, 921.9, and 918.1) were assembled. Because most of the comorbidities were too rare to be assessed, we included them only if they occurred in one or more outpatient settings, or if the condition appeared in hospitalization care claims within 1 year prior to the index date.

### Statistical Analysis

We used SAS v. 9.4 for Windows (SAS Institute, Inc., Cary, NC, USA) for all statistical analyses. The basic demographics and comorbidities between the AKC and control groups were compared using Pearson's chi-square analysis. The RCE incidence was calculated as the total numbers of RCE patients discovered during the follow-up span, divided by the whole person-years (PY) for the respective groups according to age, sex, and chosen comorbidities. The Poisson regression analysis was performed to obtain the incidence rate ratio (IRR), to compare the risk of developing RCE between the AKC patients and non-AKC controls. The Cox proportional hazards regression was applied to determine the differences in the adjusted hazard ratios (HRs) and 95% confidence intervals (CIs) for the risk of RCE. The Kaplan–Meier method was employed to construct the cumulative incidence curves and the log-rank test was applied to evaluate the differences. Statistical significance was set at *p* < 0.05.

## Results

### Demographic Data

After eliminating ineligible participants, 184,166 AKC patients and 184,166 control patients were enrolled. Table 1 reveals the initial demographics and comorbidities of both groups. The average age in both the groups was 31.95 (standard deviation, 19.42) years. The onset age of AKC was <12, 12–19, 20–29, 30–39, and ≥ 40 years in 35,110 (19.06%), 25,936 (14.08%), 34,834 (18.91%), 29,873 (16.22%), and 58,413 (31.72%) patients, respectively. Of the 184,166 patients with AKC, 76,361 (41.46%) were men and 107,805 (58.54%) were women. Regarding comorbidities, AKC patients demonstrated a significantly higher prevalence of previously reported risk factors for RCE, such as diabetes mellitus (*p* < 0.0001), keratoconjunctivitis sicca (*p* < 0.0001), and corneal transplantation (*p* = 0.0012), than the controls. There was no significant difference in prevalence of ocular blunt trauma, corneal dystrophy, and band keratopathy. The mean follow-up periods for the AKC and control group patients were 5.64 (SD, 2.28) and 5.87 (SD, 2.29) years, respectively (*p* < 0.0001).

**Table 1:** Comparison of the demographic characteristics and comorbidities between the atopic keratoconjunctivitis (AKC) and control group.

	**AKC**	**Controls**	
	**(*N* = 184,166)**	**(*N* = 184,166)**	***p*-value**
Age (years; mean ± SD)	31.95 ± 19.42	31.95 ± 19.42	1.0000
	**Number (%)**	**Number (%)**	
**Age group (years)**		
<12	35,110 (19.06)	35,110 (19.06)	1.0000
12–19	25,936 (14.08)	25,936 (14.08)	
20–29	34,834 (18.91)	34,834 (18.91)	
30–39	29,873 (16.22)	29,873 (16.22)	
≥40	58,413 (31.72)	58,413 (31.72)	
**Sex**		
Male	76,361 (41.46)	76,361 (41.46)	1.0000
Female	107,805 (58.54)	107,805 (58.54)	
**Baseline comorbidity**		
Diabetes mellitus	17,687 (9.60)	14,663 (7.96)	<0.0001
Keratoconjunctivitis sicca	7,415 (4.03)	2,595 (1.41)	<0.0001
Ocular blunt trauma	147 (0.08)	120 (0.07)	0.0983
Post-corneal transplantation	65 (0.04)	33 (0.02)	0.0012
Corneal dystrophy	80 (0.04)	70 (0.04)	0.4141
Band keratopathy	19 (0.01)	13 (0.01)	0.2888
Follow-up period (years; mean ± SD)	5.64 ± 2.28	5.87 ± 2.29	<0.0001

*The demographic characteristics and comorbidities between the AKC and control groups were compared using Pearson chi-square tests. AKC, atopic keratoconjunctivitis; SD, standard deviation*.

### Incidence of RCE

During the follow-up span, there was a higher RCE incidence in AKC patients (5.43/10,000 PY) than in matched controls (3.76/10,000 PY), leading to a significant difference in the IRR of RCE (1.45, 95% CI = 1.27–1.64, *p* <0.0001; Table 2).

**Table 2:** Risk of recurrent corneal erosion (RCE) in the atopic keratoconjunctivitis and control groups.

**Characteristics**	**Atopic keratoconjunctivitis**				**Controls**				**IRR (95% CI)**	***p*-value**
	***N***	**RCE**	**PY**	Rate^*^	***N***	**RCE**	**PY**	Rate^*^		
All	184,166	564	1,038,679	5.43	184,166	406	1,080,719	3.76	1.45 (1.27–1.64)	<0.0001
**Age (years)**										
<12	35,110	31	206,829	1.50	35,110	42	246,845	1.70	0.88 (0.55–1.40)	0.5923
12–19	25,936	66	146,643	4.50	25,936	55	146,724	3.75	1.20 (0.84–1.72)	0.3165
20–29	34,834	111	200,998	5.52	34,834	105	201,038	5.22	1.06 (0.81–1.38)	0.6821
30–39	29,873	90	166,409	5.41	29,873	59	166,702	3.54	1.53 (1.10–2.12)	0.0114
≥40	58,413	266	317,800	8.37	58,413	145	319,410	4.54	1.84 (1.51–2.26)	<0.0001
**Sex**										
Male	76,361	185	430,909	4.29	76,361	122	456,011	2.68	1.60 (1.28–2.02)	<0.0001
Female	109,025	379	607,770	6.24	107,805	284	624,708	4.55	1.37 (1.18–1.60)	<0.0001
**Comorbidity**										
Diabetes mellitus	17,687	87	93,338	9.32	14,663	49	77,000	6.36	1.47 (1.03–2.08)	0.0326
Keratoconjunctivitis sicca	7,415	49	37,983	12.90	2,59,5	13	12,401	10.48	1.23 (0.67–2.27)	0.5060
Post-corneal transplantation	65	7	337	207.72	33	2	170	117.65	1.77 (0.37–8.53)	0.4756
Ocular blunt trauma	147	2	674	29.67	120	0	617	0	-	-
Corneal dystrophy	80	0	397	0	70	1	399	25.06	-	-
Band keratopathy	19	0	90	0	13	0	72	0	-	-

*A Poisson regression analysis was performed to calculate the incidence rate ratio. IRR, incidence rate ratio; PY: person-years; RCE: recurrent corneal erosion*.

* *Rate: per 10,000 person-years*.

Patients with AKC aged ≥ 40 years showed the highest RCE incidence (8.37/10,000 PY), followed by patients aged 20–29 years (5.52/10,000 PY), 30–39 years (5.41/10,000 PY), 12–19 years (4.50/10,000 PY), and <12 years (1.50/10,000 PY). The values of the IRR were significantly higher for AKC patients than for controls in patients aged 30–39 years (1.53 [95% CI = 1.10–2.12; *p* = 0.0114]), and ≥ 40 years (1.84 [95% CI = 1.51–2.26; *p* < 0.0001]). Nevertheless, no significant difference existed in the RCE incidence between AKC patients aged <12 years, 12–19 years or 20–29 years, and their corresponding controls (Table 2). The RCE incidence was 4.29/10,000 PY for male AKC patients and 2.68/10,000 PY for male controls (IRR = 1.60; 95% CI = 1.28–2.02*; p* < 0.0001). A significant difference was also observed between female AKC patients and female controls (IRR = 1.37; 95% CI = 1.18–1.60; *p* < 0.0001; Table 2).

The RCE incidence was 9.32/10,000 PY in patients with diabetes mellitus and 6.36/10,000 PY in controls. The IRR for RCE in AKC patients with diabetes mellitus was 1.47 times higher than in controls (IRR = 1.47; 95% CI = 1.03–2.08; *p* = 0.0326). It is worth pointing out that the IRRs for RCE did not denote significantly greater risks in AKC patients with keratoconjunctivitis sicca, corneal transplantation, or ocular blunt trauma than in the corresponding controls (Table 2). The IRR for RCE in ocular blunt trauma, corneal dystrophy, or band keratopathy between AKC patients and the controls could not be determined, because so few patients with these conditions developed RCE in both groups (Table 2).

Table 3 displays the crude and adjusted HRs for RCE during the follow-up period. After adjusting for age, sex, and the selected comorbidities, AKC remained an independent risk of RCE (adjusted HR = 1.36; 95% CI = 1.19–1.54; *p* < 0.05). In both the AKC and control groups, patients aged 12–19 years (adjusted HR, 2.36; 95% CI = 1.76–3.15; *p* < 0.05), 20–29 years (adjusted HR, 2.93; 95% CI = 2.24–3.83; *p* < 0.05), 30–39 years (adjusted HR, 2.39; 95% CI = 1.80–3.17; *p* < 0.05), and 40–49 years (adjusted HR, 3.17; 95% CI = 2.45–4.11; *p* < 0.05) as well as women (adjusted HR, 1.35; 95% CI = 1.18–1.55; *p* < 0.05) were at a higher risk of developing RCE. Diabetes mellitus (adjusted HR, 1.32; 95% CI = 1.09–1.61; *p* < 0.05), keratoconjunctivitis sicca (adjusted HR, 1.85; 95% CI = 1.42–2.42; *p* < 0.05), and corneal transplantation (adjusted HR, 26.33; 95% CI = 13.55–51.18; *p* < 0.05) were significant risk factors for RCE in both groups, whereas ocular blunt trauma and corneal dystrophy were not independent risk factors for RCE. We could not appraise whether band keratopathy was a significant risk factor after adjusting for other confounding factors in the total cohort because of the lack of RCE incidence among patients with these comorbidities in both groups.

**Table 3:** Crude and adjusted hazard ratios for the Cox proportional hazard regression analyses and 95% confidence intervals for recurrent corneal erosion in the study cohort during the follow-up period.

**Cohort**	**Crude hazard ratio (95% CI)**	**Adjusted hazard ratio (95% CI)**
**Atopic keratoconjunctivitis**		
Yes	1.44^*^ (1.26–1.63)	1.36^*^ (1.19–1.54)
No	1.00	1.00
**Age (years)**		
<12	1.00	1.00
12–19	2.51^*^ (1.88–3.36)	2.36^*^ (1.76–3.15)
20–29	3.28^*^ (2.52–4.28)	2.93^*^ (2.24–3.83)
30–39	2.72^*^ (2.05–3.60)	2.39^*^ (1.80–3.17)
≥40	3.91^*^ (3.05–5.01)	3.17^*^ (2.45–4.11)
**Sex**		
Female	1.55^*^ (1.35–1.77)	1.35^*^ (1.18–1.55)
Male	1.00	1.00
**Comorbidity**		
Diabetes mellitus	1.84^*^ (1.53–2.20)	1.32^*^ (1.09–1.61)
Keratoconjunctivitis sicca	2.73^*^ (2.11–3.54)	1.85^*^ (1.42–2.42)
Post-corneal transplantation	38.63^*^ (20.04–74.47)	26.33^*^ (13.55–51.18)
Ocular blunt trauma	3.29(0.82–13.16)	3.71 (0.93–14.85)
Corneal dystrophy	2.72 (0.38–19.30)	1.67 (0.23–11.91)
Band keratopathy	-	-

*The adjusted hazard ratio for developing a recurrent corneal erosion was calculated using the Cox proportional hazard regression analysis. CI, confidence interval*.

* *p < 0.05*.

**Figure 2:**
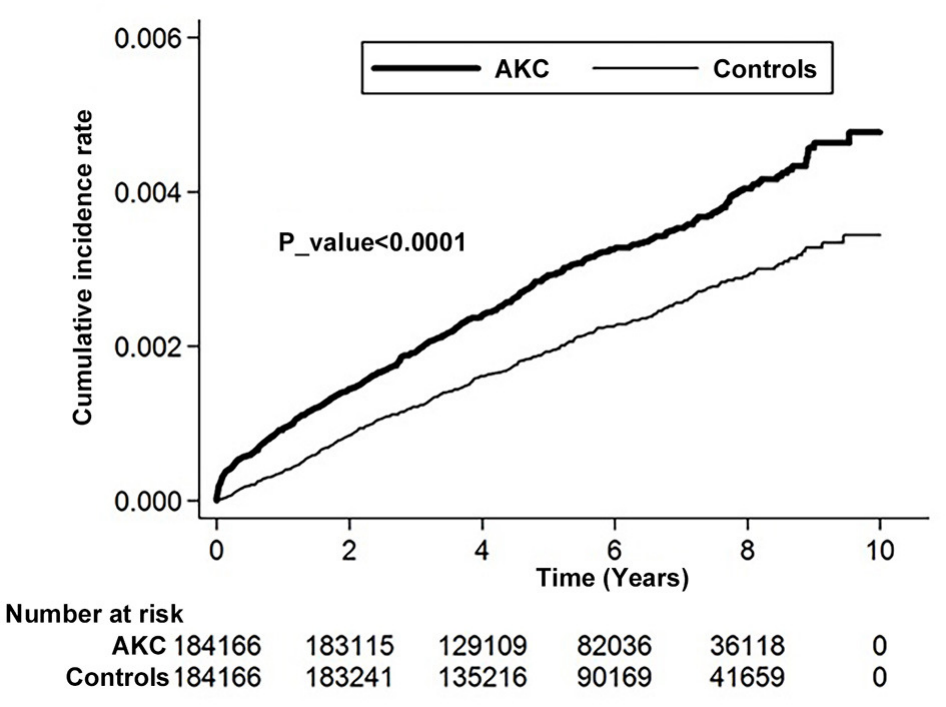
Cumulative incidence rate of recurrent corneal erosion in patients with atopic keratoconjunctivitis and controls during the follow-up period.

Kaplan–Meier analyses revealed a higher cumulative incidence of RCE in the AKC group than in the control group, and the log-rank test findings were also significant (*p* < 0.0001; Figure 2).

## Discussion

To the best of our knowledge, our study is the most large-scale population-based investigation to examine the relation between AKC and RCE. We investigated 184,166 AKC patients and 184,166 controls and discovered that the RCE incidence in AKC patients was 1.45 times higher than that in controls, and the relative risk of developing RCE in AKC patients increased 1.36 times in the whole cohort after adjustment for age, sex, diabetes mellitus, keratoconjunctivitis sicca, corneal transplantation, ocular blunt trauma, corneal dystrophy, and band keratopathy.

The association between AKC and RCE has been discussed in a few previous reports (10, 11). Messmer et al. (11) evaluated three corneal buttons of a patient with AKC having RCE or ulcerations, and detected a linear subepithelial deposition of eosinophil granular substances such as major basic protein and eosinophil cationic protein above the Bowman's membrane in all corneal buttons. They proposed that toxic eosinophil proteins may be involved in the pathophysiology of RCE in patients with AKC. Fukagawa collected tear samples from 38 eyes of 24 patients with AKC with different corneal conditions including RCE, superficial punctate keratopathy, and no additional pathology, and found significantly high concentrations of eotaxin and eosinophilic infiltration in the tears of patients with AKC with RCE (10). It is worth noting that eosinophils are known to be responsible for corneal damage through the release of toxic mediators (15). In addition, eosinophils play an pathogenic role in ocular surface disorders in AKC patients, and the clinical status of ocular surface disorders of AKC patients is associated with the tear eosinophil cationic protein levels (16–18). The high concentration of eosinophil granular substances in patients with AKC may be a possible explanation for the association between AKC and RCE.

Frequent eye rubbing is a common physiologic response to fatigue, itchy sensation, and discomfort in the eyes in AKC patients (19). Prolonged and vigorous eye rubbing may lead to an ocular surface injury in AKC patients, which is a possible risk factor for the development of RCE. Besides mechanical trauma to the ocular surface due to eye rubbing, inflammatory processes following eye rubbing have been found in patients with AKC (19). Several reports have shown that inflammatory mediators, such as interleukin (IL)-1, IL-6, and tumour necrosis factor -α, are higher in the tears of AKC patients than in those of healthy controls (20–22). The release of inflammatory cytokines, including IL-1 and IL-6, is also triggered by corneal injuries, and leads to corneal basement membrane disruption and weakening of the extra-cellular adhesion network (8, 9). Although these cytokines play an important role in wound healing by up-regulation of the integrin receptor for fibronectin or over-expression of keratinocyte growth factor, and a synergistic effect with epithelial growth factor during the wound healing process, a balance between healing and disruption has to be maintained (23–25). Candar et al. (8) reported that the levels of IL-1 and IL-6 were increased in patients with RCE when compared with healthy controls. In addition, they showed that the positive correlation between the levels of these cytokines and epithelial growth factor in healthy controls was disrupted in patients with RCE. The elevated IL-1 and IL-6 in AKC patients possibly aggravate the imbalance, which could elucidate the causal association between AKC and the later development of RCE.

Several studies have presented that MMPs, particularly MMP-2 and MMP-9, were upregulated by inflammatory events in patients with AKC (19, 22, 26). The higher concentration of MMP in the tears of patients with AKC than in those of healthy controls has been reported by several studies (22, 26, 27). It is worth noting that MMP-2 and MMP-9 are also highly expressed in the corneal epithelium and tears of patients with RCE (13, 14). Enhanced expression of MMP in patients with RCE promotes cleavage of adhesion molecules and collagens leading to hemi-desmosome dysfunction (12, 14). These findings with regards to the upregulation of MMPs may imply a causal relationship between AKC and RCE.

We found that the incidence of RCE was higher in women with AKC (Table 2). The risk of developing RCE is 1.35 times higher in female patients than in male patients (Table 3). This finding can be possibly explained by the higher probability of development of RCE in women, demonstrated by most of the major studies (28–30).

Several studies have shown that comorbidities, such as diabetes mellitus (7, 31), keratoconjunctivitis sicca (6, 31, 32), corneal transplantation (9, 33), ocular blunt trauma (9, 33), corneal dystrophy, and band keratopathy are associated with RCE. In this cohort study, we evaluated these comorbidities in AKC patients and discovered that AKC patients with diabetes mellitus had a significantly higher IRR for RCE than the controls (Table 2), and diabetes mellitus was a significant risk factor for the development of RCE in the whole cohort (Table 3). This finding is consistent with several previous studies (6, 7, 31). Nanba et al. (7) conducted a retrospective study including 21 eyes of 21 patients with RCE and found that diabetes mellitus is an important risk factor for RCE. Our previous retrospective, nationwide, matched cohort study that included 239,854 patients with diabetes mellitus showed that patients with diabetes mellitus were 1.35 times (95% CI, 1.24–1.48) more likely to develop RCE than the total sample cohort (31). The association between diabetes mellitus and RCE may be explained by the influence of hyperglycaemia in patients with diabetes mellitus on corneal morphology, and physiology including delayed epithelial wound healing, reduced basal epithelial cell density, and decreased sub-basal nerve density in the cornea of patients with diabetes mellitus (34).

Further, we found that keratoconjunctivitis sicca is an independent risk factor for the development of RCE in the total cohort (Table 3), although keratoconjunctivitis sicca did not lead to a significantly higher RCE incidence in the AKC patients and controls (Table 2). The association between keratoconjunctivitis sicca and RCE has been reported in several papers (31, 32). Our previous study showed the relationship between keratoconjunctivitis sicca and RCE in the total cohort of 239,854 patients with diabetes mellitus and 239,854 matched control (31). Keratoconjunctivitis sicca, characterised by inflammation and a loss of homeostasis of the tear film, is a complex ocular surface disorder and a multifactorial condition accompanied by many ocular symptoms, including discomfort, visual disturbance, and foreign body sensation (35). The most important pathophysiology of keratoconjunctivitis sicca is a vicious cycle including lacrimal gland destruction, corneal sensory nerve damage, and reduced tear production (36). The association between keratoconjunctivitis sicca and RCE may be due to the common pathophysiological mechanisms.

There are several vantages in our study. First, our large-scale cohort study had great precision of risk appraisal and statistical power because of a large number of enrolees, including 184,166 AKC patients and 184,166 controls. Moreover, the potential confounding bias was reduced, by adjusted for diabetes mellitus, keratoconjunctivitis sicca, corneal transplantation, ocular blunt trauma, corneal dystrophy, and band keratopathy in our cohort study with longitudinal data of up to 10 years.

This study also had limitations. The history of AKC before January 1996 in the controls could not be verified, because the medical history of each participant in the database could only be traced back to 1996. Additionally, band keratopathy, a well-known important confounding factor, could not be evaluated truly because no band keratopathy developed RCE in the AKC and control groups. Moreover, some important confounders including the use of contact lenses, mild ocular trauma, and record of ocular refractive surgery were not assessed. Finally, the diagnosis of AKC, RCE, and other diseases depended on ICD-9-codes, so incorrect classification was possible.

In brief, this study revealed that AKC patients have a significantly higher risk of developing RCE. AKC remained an independent risk factor after adjustment for confounders in the total cohort. These results indicate that clinicians should notify AKC patients of their increased RCE risk.

## Data Availability Statement

The original contributions presented in the study are included in the article/supplementary material, further inquiries can be directed to the corresponding author/s.

## Ethics Statement

The requirements for ethical approval and informed consent were waived by the Institutional Review Board of the Chi-Mei Medical Center, because no identifiable personal information was analysed using the public database.

## Author Contributions

R-LJ and Y-SC: conceptualization. R-LJ, S-FW, and Y-SC: formal analysis, methodology, and writing—original draft. J-JW: resources and software. S-HT and Y-SC: writing—review and editing. All authors contributed to the article and approved the submitted version.

## Conflict of Interest

The authors declare that the research was conducted in the absence of any commercial or financial relationships that could be construed as a potential conflict of interest.

## Abbreviations

AKC: atopic keratoconjunctivitis
CI: confidence interval
HR: hazard ratio
ICD-9-CM, International Classification of Diseases, Ninth Revision: Clinical Modification
IL: interleukin
IRR: incidence rate ratio
LHID 2000: Longitudinal Health Insurance Database 2000
MMP: matrix metalloproteinase
NHIRD: National Health Insurance Research Database
NHRI: National Health Research Institute
PY: person-years
RCE: recurrent corneal erosion
SD: standard deviation.
